# Can circulating PD-1, PD-L1, BTN3A1, pan-BTN3As, BTN2A1 and BTLA levels enhance prognostic power of CA125 in patients with advanced high-grade serous ovarian cancer?

**DOI:** 10.3389/fonc.2022.946319

**Published:** 2022-09-21

**Authors:** Daniele Fanale, Lidia Rita Corsini, Chiara Brando, Sofia Cutaia, Mariano Catello Di Donna, Clarissa Filorizzo, Maria Chiara Lisanti, Ugo Randazzo, Luigi Magrin, Raffaella Romano, Tancredi Didier Bazan Russo, Daniel Olive, Salvatore Vieni, Gianni Pantuso, Vito Chiantera, Antonio Russo, Viviana Bazan, Juan Lucio Iovanna

**Affiliations:** ^1^ Section of Medical Oncology, Department of Surgical, Oncological and Oral Sciences, University of Palermo, Palermo, Italy; ^2^ Department of Gynecologic Oncology, University of Palermo, Palermo, Italy; ^3^ Team Immunity and Cancer, Centre de Recherche en Cancérologie de Marseille (CRCM), Institut National de la Santé et de la Recherche Médicale (INSERM) U1068, Centre National de la Recherche Scientifique Unité Mixte de Recherche (CNRS UMR) 7258 Aix-Marseille Université and Institut Paoli-Calmettes, Marseille, France; ^4^ Division of General and Oncological Surgery, Department of Surgical, Oncological and Oral Sciences, University of Palermo, Palermo, Italy; ^5^ Department of Biomedicine, Neuroscience and Advanced Diagnostics, University of Palermo, Palermo, Italy; ^6^ Centre de Recherche en Cancérologie de Marseille (CRCM), Institut National de la Santé et de la Recherche Médicale (INSERM) U1068, Centre National de la Recherche Scientifique Unité Mixte de Recherche (CNRS UMR) 7258, Aix-Marseille Université and Institut Paoli-Calmettes, Parc Scientifique et Technologique de Luminy, Marseille, France

**Keywords:** BTLA, butyrophilins, serum CA125, circulating immune checkpoints, HGSOC, PD-1, PD-L1, prognostic factors

## Abstract

The most common subtype of ovarian cancer (OC) is the high-grade serous ovarian carcinoma (HGSOC), accounting for 70%–80% of all OC deaths. Although HGSOC is a potentially immunogenic tumor, clinical studies assessing the effectiveness of inhibitors of programmed death protein and its ligand (PD-1/PD-L1) in OC patients so far showed only response rates <15%. However, recent studies revealed an interesting prognostic role of plasma PD-1/PD-L1 and other circulating immunoregulatory molecules, such as the B- and T-lymphocyte attenuator (BTLA), butyrophilin sub-family 3A/CD277 receptors (BTN3A), and butyrophilin sub-family 2 member A1 (BTN2A1), in several solid tumors. Since evidence showed the prognostic relevance of pretreatment serum CA125 levels in OC, the aim of our study was to investigate if soluble forms of inhibitory immune checkpoints can enhance prognostic power of CA125 in advanced HGSOC women. Using specific ELISA tests, we examined the circulating PD-1, PD-L1, pan-BTN3As, BTN3A1, BTN2A1, and BTLA levels in 100 advanced HGSOC patients before treatment, correlating them with baseline serum CA125, age at diagnosis, body mass index (BMI), and peritoneal carcinomatosis. A multivariate analysis revealed that plasma BTN3A1 ≤4.75 ng/ml (HR, 1.94; 95% CI, 1.23–3.07; p=0.004), age at diagnosis ≤60 years (HR, 1.65; 95% CI, 1.05–2.59; p=0.03) and absence of peritoneal carcinomatosis (HR, 2.65; 95% CI, 1.66–4.22; p<0.0001) were independent prognostic factors for a longer progression-free survival (PFS) (≥30 months) in advanced HGSOC women. However, further two-factor multivariate analyses highlighted that baseline serum CA125 levels >401 U/ml and each soluble protein above respective concentration cutoff were covariates associated with shorter PFS (<30 months) and unfavorable clinical outcome, suggesting that contemporary measurement of both biomarkers than CA125 only could strengthen prognostic power of serum CA125 in predicting PFS of advanced HGSOC women. Plasma PD-L1, PD-1, BTN3A1, pan-sBTN3As, BTN2A1, or BTLA levels could be helpful biomarkers to increase prognostic value of CA125.

## Introduction

Ovarian cancer (OC) is the seventh most frequently diagnosed tumor and the eighth leading cause of cancer death in women worldwide, with a 5-year relative survival rate of 49% (1, 2).

High-grade serous ovarian carcinoma (HGSOC) is the most recurrent subtype and represents 70%–80% of all OC deaths (3). Unfavorable prognosis of HGSOC is determined by tumor heterogeneity and therapy resistance (4). Standard treatment for OC includes surgery and platinum-based chemotherapy (5). Improvements in the progression-free survival (PFS) and overall survival (OS) were achieved by neoadjuvant chemotherapy followed by interval debulking surgery (3). Nevertheless, recurrence rate still remains elevated and about 70% of women with advanced OC relapses with a worse prognosis (6, 7). Immunotherapy, whose effectiveness was demonstrated in other tumors, including non-small cell lung cancer (8, 9), renal cell carcinoma (10, 11), and melanoma (12), has not yielded the expected results in OC (13), despite the presence of tumor-infiltrating lymphocytes (TILs) (14–16).

The most investigated immune checkpoint receptor is the programmed cell death protein 1 (PD-1), with its ligands, PD-L1, and PD-L2 (17). Although PD-L1 expression was detected in more than 50% of advanced OCs, early-phase trials on effectiveness of anti-PD-1/PD-L1 agents exhibited an overall response rate (ORR) between 8% and 60% and a median PFS of 2–10 months (4, 18).

Other immune checkpoints, including B- and T-lymphocyte attenuator (BTLA) (19), butyrophilin sub-family 3 member A1 (BTN3A1) receptor, pan-BTN3A, and butyrophilin sub-family 2 member A1 (BTN2A1) (20), showed an interesting immunomodulatory role in different tumors (11, 21, 22). The activation of these immunoregulatory molecules (including PD-1 and PD-L1) able to positively or negatively modulate anti-tumor immune response may allow, in some cases, cancer cells to overcome immune surveillance (23).

Recently, new evidence showed that investigating the soluble forms of inhibitory immune checkpoints may allow to obtain useful information about the evolution of cancer by predicting survival of patients affected by various tumors. Therefore, these studies suggested their potential use as prognostic biomarkers (11, 21, 22, 24–26). Since plasma is a biological sample that can be easily obtained with little invasiveness, evaluating the plasma concentrations of inhibitory immune checkpoints may provide us a more dynamic profile of the tumor microenvironment and a better overview of disease by overcoming the limitations arising from tissue biopsy (invasiveness, limited quantity of sample, and poor dynamism) (22).

Since evidence showed the potential prognostic relevance of pretreatment serum CA125 (Cancer antigen 125) levels in OC (27), the aim of our study was to investigate if soluble forms of inhibitory immune checkpoints, such as PD-1 (sPD-1), PD-L1 (sPD-L1), BTN3A1 (sBTN3A1), pan-BTN3As (pan-sBTN3As), BTN2A1 (sBTN2A1), and BTLA (sBTLA), can act as useful biomarkers to enhance prognostic power of serum CA125 in advanced HGSOC women.

## Patients and methods

### Study cohort

We prospectively studied a cohort of 100 advanced HGSOC women enrolled at the two Sicilian hospital centers: “Sicilian Regional Center for the Prevention, Diagnosis and Treatment of Rare and Heredo-Familial Tumors” of the Section of Medical Oncology of University Hospital Policlinico “P. Giaccone” of Palermo (Italy) and Department of Gynecologic Oncology of the Hospital ARNAS Civico “Di Cristina Benfratelli” of Palermo (Italy).

The study (Protocol “TIC-OC v.1.1”) was approved by the ethical committee (Comitato Etico Palermo 1) of the university-affiliated hospital A.O.U.P. “P. Giaccone” of Palermo (Italy).

The information regarding the age at diagnosis, personal history, histological subtype, grading, and International Federation of Gynecology and Obstetrics (FIGO) stages were anonymously collected for all recruited patients (**Table 1**) who had previously signed a written informed consent.

**Table 1:** Clinical and pathological characteristics of advanced HGSOC patients.

Characteristic	No. of Patients (%)
Total patients	*100*
**Age at diagnosis (y):** Median: 61 Mean: 60 Range: 27-79	
**Age groups (y)** ≤ 60 > 60	48 (48) 52 (52)
FIGO stage **^a^** IIIB IIIC IV	23 (23) 52 (52) 25 (25)
**Histological grade** G1/2 G3	0 (0) 100 (100)
**Histological subtype** Serous Other	100 (100) 0 (0)
**OC** Unilateral Bilateral	64 (64) 36 (36)
**Surgery** Surgical staging Cytoreductive surgery	52 (52) 48 (48)
**Serum CA125 levels** ≤ 401 > 401	50 (50) 50 (50)
**Peritoneal carcinomatosis** Yes No	43 (43) 57 (57)
**BMI** ≤ 25 > 25	59 (59) 41 (41)
**Smoker** Yes No	23 (23) 77 (77)

a AJCC Cancer Staging Manual 8th staging.

BMI, body mass index; CA125, cancer antigen 125; FIGO, International Federation of Gynecology and Obstetrics.

From May 2018 to July 2021, blood specimens were prospectively harvested from 100 patients with histological diagnosis of advanced HGSOC (stage IIIB–IV) at baseline, before surgery (surgical staging or cytoreductive surgery as clinically recommended), and starting first-line chemotherapy with Carboplatin AUC (area under the curve) 5 and Paclitaxel (175 mg/m^2^) according to the current therapeutic strategies. Patients with Eastern Cooperative Oncology Group (ECOG) Performance Status (PS) ≥3 were excluded from the study.

An independent validation cohort of 24 advanced HGSOC women, enrolled at the Section of Medical Oncology of University Hospital Policlinico “P. Giaccone” of Palermo (Italy), was used to confirm the previously obtained data.

### Analysis of plasma PD-1, PD-L1, BTN3A1, pan-BTN3As, BTN2A1, and BTLA dosages

Baseline peripheral blood specimens from untreated advanced HGSOC women were collected, processed for plasma isolation, and analyzed through specific enzyme-linked immunosorbent assays (ELISAs) as previously described (10, 11, 28) to determine plasma concentrations of sPD-1, sPD-L1, sBTN3A1, pan-sBTN3As, sBTN2A1, and sBTLA. In this analysis, the soluble forms of all six immune checkpoints were detected in the plasma rather than in the serum because serum concentrations have been shown to be 10 times lower than those detected in the plasma from the same blood sample. Probably, most of the tested biomarkers were apparently lost due to the clotting process. For this reason, only plasma samples were used in our investigation. Furthermore, a dilution of all samples in the ratio of 1–5 was performed before running the ELISAs in order to prevent interference processes due to the plasma matrix.

Since some discrepancies, concerning the performances, reproducibility, sensitivity and specificity, cross-reactivity, differences in quantification in the plasma and serum, and run temperature, were observed in other commercially available tests, specific ELISAs produced by the company DYNABIO S.A. (Parc de Luminy, Marseille, France) were used according to the previously described recommendations (21, 22). All specifications concerning the features of six ELISAs are reported in **Supplementary Table S1**.

In particular, these specific ELISAs were used because some assays were either not commercially available (tests for pan-BTN3A, BTN3A1, and BTN2A1) or were not satisfactory (lack of sensitivity, specificity, or reproducibility in our own preliminary studies). These ELISA tests not only showed very good performances but also established conditions for optimal determination of concentrations of the six markers in blood (use of plasma instead of serum), solving the problem regarding the differences in quantification of proteins between the plasma and serum, which are detected when using other commercial kits. All six used ELISA tests showed good linearity and a high specificity. The linearity for sPD-1 measurement in the test ranges from 0.01 to 5.00 ng/ml, for sPD-L1 from 0.02 to 2.00 ng/ml, for pan-sBTN3As from 0.10 to 8.00 ng/ml, for sBTN3A1 from 0.05 to 8.00 ng/ml, for sBTN2A1 from 0.03 to 2.00 ng/ml, and for sBTLA from 0.25 to 8.00 ng/ml, as shown in **Supplementary File 1**. In addition, we tested the cross-reactivity between these six recombinant proteins, and, as expected, no signal was detected when the antibodies used did not correspond to the antigen.

Specific details on the experimental protocol regarding the used ELISA assays are reported in **Supplementary File 2**.

### Data analysis

An analysis by receiver operating characteristic (ROC) curves (29) was carried out to identify the optimal concentration thresholds for each soluble form of immune checkpoints and other examined clinicopathological factors (CA125, age at diagnosis, and BMI) in order to divide HGSOC women based on long (≥30 months) versus short PFS (<30 months). The Kaplan–Meier method and log-rank test were applied to perform association analysis of biomarkers and other factors with PFS. We used univariate and multivariate Cox proportional hazard regression models to identify significant prognostic factors for PFS (22). MedCalc software v.18.2.1 for Windows (MedCalc Software, Ostend, Belgium) and GraphPad Prism software v. 9.0.0 (GraphPad Software, San Diego, CA) were used to generate and represent data (22). p-values <0.05 were considered statistically significant.

## Results

### Determination of the optimal thresholds to discriminate long versus short PFS advanced HGSOC patients

Using specific ELISAs, we performed the measurement of plasma levels of sPD-1, sPD-L1, sBTN3A1, pan-sBTN3As, sBTN2A1, and sBTLA in 100 advanced HGSOC women, before surgery and of starting first-line chemotherapy.

The optimal concentration cutoffs (Youden-index-associated criterion) to discriminate advanced HGSOC patients based on long (≥30 months) versus short PFS (<30 months) were determined for each circulating immune checkpoint through ROC analysis.

The best concentration cutoffs were 2.48 ng/ml for sPD-1 (AUC=0.60, p=0.04), 0.42 ng/ml for sPD-L1 (AUC=0.71, p=0.01), 4.75 ng/ml for sBTN3A1 (AUC=0.64, p=0.01), 13.06 ng/ml for pan-sBTN3As (AUC=0.65, p=0.008), 5.59 ng/ml for sBTN2A1 (AUC=0.64, p=0.02), and 2.78 ng/ml for sBTLA (AUC=0.62, p=0.02). The same analysis also allowed to establish the most suitable thresholds of three different considered factors: age at diagnosis, baseline CA125, and BMI. Therefore, the optimal thresholds for age at diagnosis, CA125, and BMI were, respectively, the following: 60 years (AUC=0.67, p=0.002), 401 U/ml (AUC=0.59, p=0.05), and 25 kg/m2 (AUC=0.62, p=0.01).

**Figure 1:**
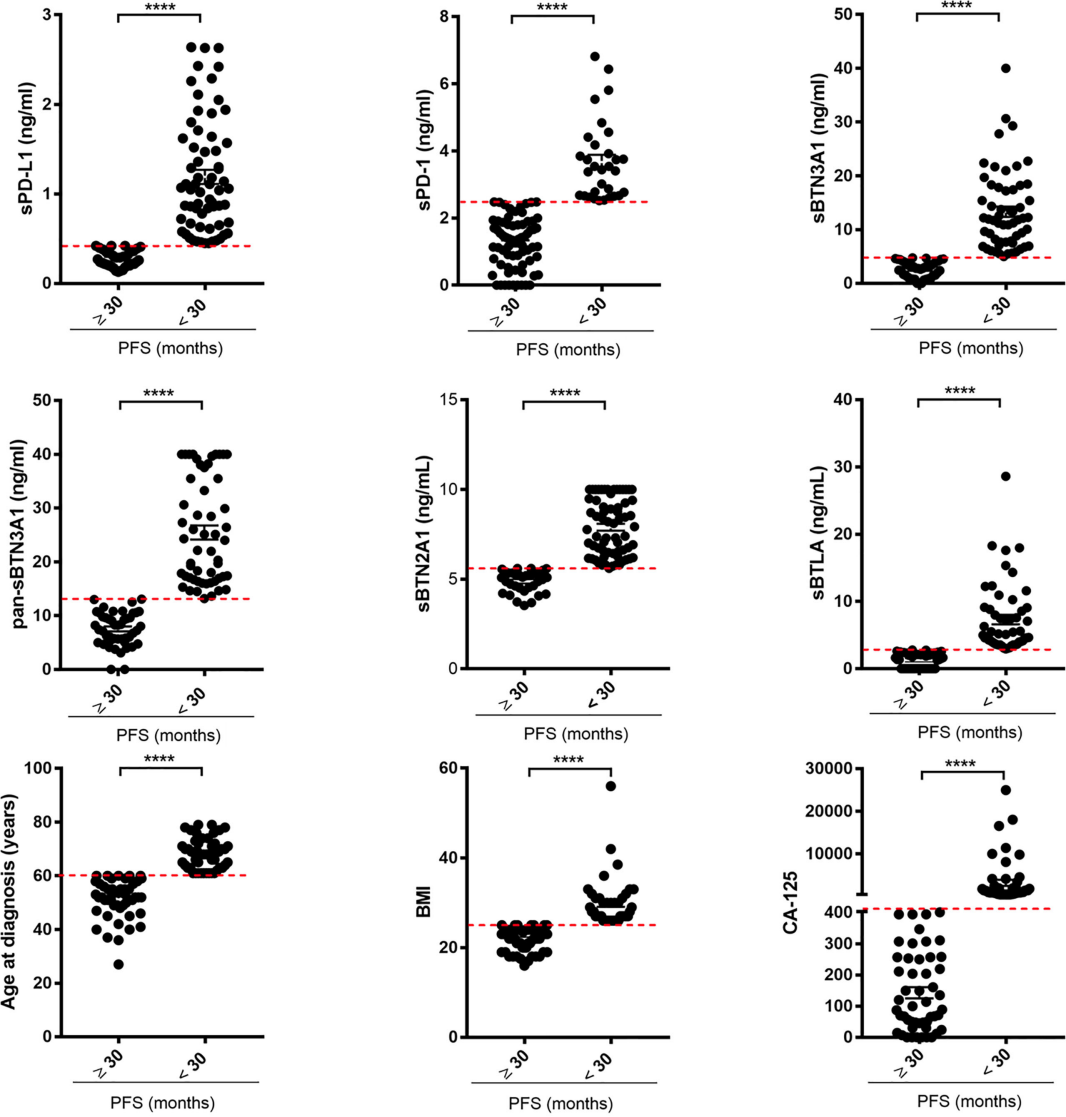
Scatter plots by group discriminating advanced HGSOC patients based on long versus short PFS for each examined factor. The plasma levels of each soluble protein, ages at diagnosis, BMIs, and baseline serous CA125 levels of advanced HGSOC patients were plotted for short (<30 months) versus long PFS (≥30 months). For each considered factor, the red dashed lines represent the optimal thresholds previously calculated by ROC analysis. The concentrations of each biomarker are reported in ng/ml. BMI, body mass index; CA125, cancer antigen 125; PFS, progression-free survival. ****p<0.0001.

Using scatter plots by group, we graphically depicted the circulating levels of each immune checkpoint, ages at diagnosis, serum CA125 levels, and BMIs, dividing the advanced-stage HGSOC women into two groups at long versus short PFS based on each examined parameter (**Figure 1**).

As shown, most of advanced HGSOC women with PFS <30 months had higher plasma levels of biomarkers (above specific thresholds) and age at diagnosis >60 years, CA125 >401 U/ml, and BMI >25.

### Low circulating levels of sPD-1, sPD-L1, sBTN3A1, pan-sBTN3As, sBTN2A1, and sBTLA correlate with a longer PFS in advanced HGSOC women

**Figure 2:**
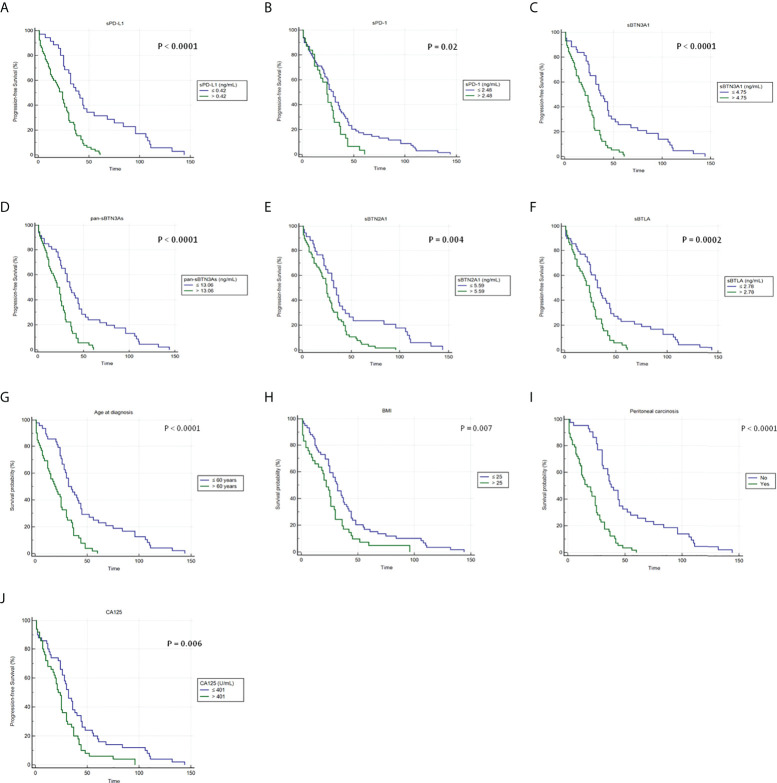
Kaplan–Meier analysis of progression-free survival in 100 advanced HGSOC patients with high and low plasma levels of **(A)** sPD-L1, **(B)** sPD-1, **(C)** sBTN3A1, **(D)** pan-sBTN3As, **(E)** sBTN2A1, and **(F)** sBTLA. In addition, Kaplan–Meier analyses showing the correlations between PFS and **(G)** age at diagnosis, **(H)** BMI, **(I)** presence of peritoneal carcinomatosis, and **(J)** baseline CA125 levels are shown. BMI, body mass index; CA125, cancer antigen 125.

Since the clinical role of plasma immune checkpoints in predicting survival of advanced HGSOC women has yet to be elucidated, we carried out a Kaplan–Meier survival analysis in order to investigate the prognostic relevance of plasma sPD-1, sPD-L1, sBTN3A1, pan-sBTN3As, sBTN2A1, and sBTLA. The thresholds previously identified by ROC analysis allowed to distinguish advanced HGSOC patients on the basis of low and high plasma levels for each analyzed marker (below and above the specific cutoffs). Kaplan–Meier curves showed the relationship between plasma concentrations of immune checkpoints and PFS (**Figures 2A–F**).

Concentration cutoffs associated with favorable prognosis and longer PFS were determined for sPD-1 (≤2.48 ng/ml), sPD-L1 (≤0.42 ng/ml), sBTN3A1 (≤4.75 ng/ml), pan-sBTN3As (≤13.06 ng/ml), sBTN2A1 (≤5.59 ng/ml), and sBTLA (≤2.78 ng/ml) (**Figures 2A–F**). Instead, patients with plasma levels above established cutoffs exhibited a median PFS, which was from 6 to 16 months shorter compared to women with levels below the concentration cutoffs.

In particular, women with lower baseline concentrations showed the following median PFS values than those with higher levels: 30 versus 24 months for sPD-1 (95% CI, 24–36 *vs*. 17–30; log-rank p-value = 0.02); 40 versus 24 months for sPD-L1 (95% CI, 30–55 *vs*. 14–28; log-rank p-value <0.0001); 37 versus 21 months for sBTN3A1 (95% CI, 32–45 *vs*. 15–26; log-rank p-value <0.0001); 35 versus 21 months for pan-sBTN3As (95% CI, 30–45 *vs*. 15–26; log-rank p-value <0.0001); 32 versus 25 months for sBTN2A1 (95% CI, 24–41 *vs*. 20–29; log-rank p-value =0.004); and 32 versus 24 months for sBTLA (95% CI, 25–44 *vs*. 17–28; log-rank p-value =0.0002).

Interestingly, baseline levels of sPD-1, sBTN2A1, and sBTLA below their specific cutoffs showed a lower benefit in median PFS (6–8 months), while a greater advantage in median PFS (14–16 months) was associated with baseline plasma concentrations of sPD-L1, sBTN3A1, and pan-sBTN3As below their specific thresholds.

Furthermore, Kaplan–Meier analysis allowed to evaluate the association between PFS and serum CA125 levels, age at diagnosis, baseline BMI, or peritoneal carcinomatosis at onset (**Figures 2G–J**).

Advanced HGSOC women with age at diagnosis >60 years, serum CA125 >401 U/ml, BMI >25, or presence of peritoneal carcinomatosis showed shorter PFS and poor prognosis. Instead, a longer median PFS (from 10 to 21 months higher) was associated with age at diagnosis ≤60 years, serum CA125 ≤401 U/ml, BMI ≤25, or absence of peritoneal carcinomatosis (**Figures 2G–J**).

Specifically, median PFS values for patients with age at diagnosis ≤60 years, serum CA125 ≤ 401 U/ml, BMI ≤25, or absence of peritoneal carcinosis compared to values above the specific thresholds were the following: 32 versus 19 months for age at diagnosis (95% CI, 28–44 *vs*. 13–25; log-rank p-value <0.0001); 32 versus 22 months for serum CA125 (95% CI, 26–38 *vs*. 18–26; log-rank p-value =0.006) and for BMI (95% CI, 25–37 *vs*. 15–26; log-rank p-value = 0.007), respectively; and 38 versus 17 months for peritoneal carcinomatosis (95% CI, 31–45 *vs*. 12–24; log-rank p-value <0.0001). No peritoneal carcinosis at diagnosis showed a greater gain in PFS.

### Plasma BTN3A1 levels, age at diagnosis, and peritoneal carcinomatosis are independent prognostic factors for PFS in advanced HGSOC women

Following the previously obtained results, we carried out a multivariate analysis for PFS to correlate the circulating PD-1, PD-L1, pan-BTN3As, BTN3A1, BTN2A1, and BTLA levels with other clinicopathological factors, such as age at diagnosis, serum CA125 levels, baseline BMI, and peritoneal carcinomatosis. Cox proportional hazard regression models were used for univariable and multivariable analyses in order to evaluate the prognostic significance of all examined parameters (**Table 2**). The univariable analyses showed a significant association between PFS and age at diagnosis, pre-treatment serum CA125 levels, baseline BMI, peritoneal carcinomatosis at onset, and plasma concentrations of sPD-1, sPD-L1, sBTN3A1, pan-sBTN3As, sBTN2A1, and sBTLA. Conversely, the final multivariable Cox regression model highlighted that only the plasma concentration of sBTN3A1>4.75 ng/ml (HR, 1.94; 95% CI, 1.23–3.07; p =0.004), age at diagnosis >60 years (HR, 1.65; 95% CI, 1.05–2.59; p = 0.03), presence of peritoneal carcinomatosis (HR, 2.65; 95% CI, 1.66–4.22; p <0.0001) were statistically significant. The other studied parameters did not show any statistically significant association. Thus, circulating sBTN3A1 ≤4.75 ng/ml (HR, 1.94; 95% CI, 1.23–3.07; p=0.004), age at diagnosis ≤60 years (HR, 1.65; 95% CI, 1.05–2.59; p=0.03), and absence of peritoneal carcinomatosis (HR, 2.65; 95% CI, 1.66–4.22; p<0.0001) have been shown to be independent prognostic factors for a longer PFS (≥30 months) in advanced HGSOC patients.

**Table 2:** Univariate and multivariate analysis of biomarkers and other factors for PFS in advanced HGSOC patients.

Factor/biomarker	Univariate Cox regression		Multivariable Cox regression	
	HR (95% CI)	p*-*value	HR (95% CI)	p*-*value
Age at diagnosis (>60 *vs*. ≤60 years)	2.57 (1.66–3.98)	<0.0001	1.65 (1.05–2.59)	0.03
Serum CA125 (>401 *vs*. ≤401 U/ml)	1.75 (1.16–2.65)	0.008	–	NS
BMI (>25 *vs*. ≤25 kg/m^2^)	1.73 (1.14–2.61)	0.007	–	NS
Peritoneal carcinosis (yes *vs*. no)	2.28 (1.51–3.45)	0.0001	2.65 (1.66–4.22)	<0.0001
sPD-L1 (>0.42 *vs*. ≤0.42 ng/ml)	3.01 (1.85–4.89)	<0.0001	–	NS
sPD-1 (>2.48 *vs*. ≤2.48 ng/ml)	1.62 (1.04–2.50)	0.02	–	NS
sBTN3A1 (>4.75 *vs*. ≤4.75 ng/ml)	2.74 (1.75–4.30)	<0.0001	1.94 (1.23–3.07)	0.004
pan-sBTN3As (>13.06 *vs*. ≤13.06 ng/ml)	2.53 (1.63–3.94)	<0.0001	–	NS
sBTN2A1 (>5.59 *vs*. ≤5.59 ng/ml)	1.92 (1.22–3.03)	0.004	–	NS
sBTLA (>2.78 *vs*. ≤2.78 ng/ml)	2.18 (1.41–3.36)	0.0002	–	NS

BMI, Body Mass Index; CA125, Cancer antigen 125; HR, Hazard Ratio; NS, Not Significant.

However, further two-factor multivariate analyses revealed that each circulating immune checkpoint (with levels above concentration cutoffs) individually correlated in a statistically significant way with baseline serum CA125 >401 U/ml levels, suggesting shorter PFS (<30 months) and poor prognosis (**Supplementary Table S2**).

### Validation analysis

A further independent cohort of 24 peripheral blood samples from advanced HGSOC women was studied to validate the previously obtained results. A Kaplan–Meier survival analysis was carried out using the same concentration cutoffs adopted for leading cohort.

**Figure 3:**
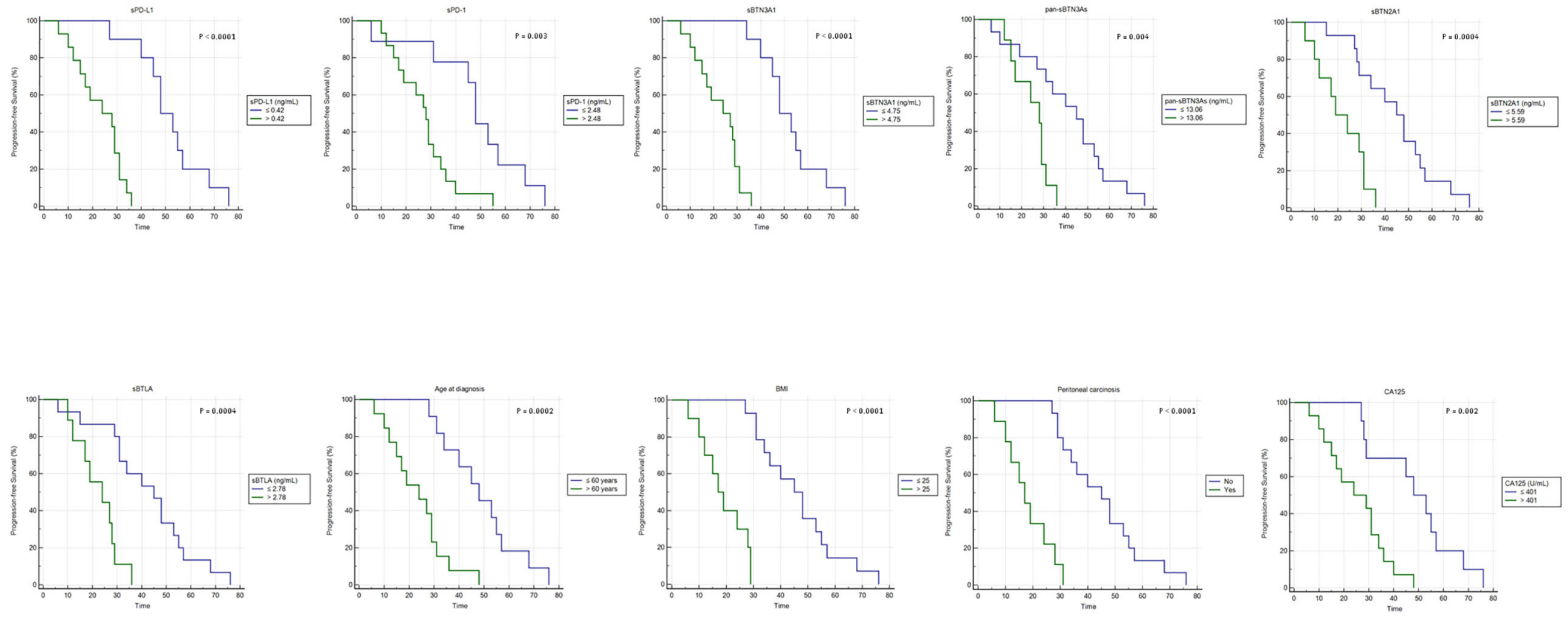
Kaplan–Meier analysis of progression-free survival in 24 advanced HGSOC patients from validation cohort. BMI, body mass index.

As previously observed, a significant inverse association between PFS and high plasma concentrations for each analyzed biomarker/factor was detected (**Figure 3**). This confirms and emphasizes our previous data obtained for the leading cohort (**Figure 2**).

In particular, advanced HGSOC women from validation cohort with lower baseline concentrations of each soluble protein exhibited the following median PFS values compared to those with higher concentrations: 48 versus 28 months for sPD-1 (95% CI, 45–57 *vs*. 19–31; log-rank p-value=0.03); 48 versus 24 months for sPD-L1 (95% CI, 45–57 *vs*. 15–31; log-rank p-value <0.0001) and sBTN3A1 (95% CI, 45–57 *vs*. 15–29; log-rank p-value <0.0001), respectively; 45 versus 28 months for pan-sBTN3As (95% CI, 31–53 *vs*. 17–29; log-rank p-value=0.004); 45 versus 19 months for sBTN2A1 (95% CI, 29–55 *vs*. 12–31; log-rank p-value = 0.0004); and 45 versus 24 months for sBTLA (95% CI, 31–53 *vs*. 17–28; log-rank p-value=0.0004).

Furthermore, an additional Kaplan–Meier analysis confirmed also for validation cohort that age at diagnosis ≤60 years, baseline serum CA125 ≤401 U/ml levels, BMI ≤25, or absence of peritoneal carcinomatosis were associated with a longer PFS (**Figure 3**). Particularly, advanced HGSOC patients with age at diagnosis ≤60 years or serum CA125 ≤401 U/ml showed a median PFS of 48 months versus 24 months of women with tumor diagnosed over 60 years of age (95% CI, 34–57 *vs*. 15–29; log-rank p-value=0.0002) or serum CA125 >401 U/ml (95% CI, 29–57 *vs*. 15–34; log-rank p-value=0.002). Women with BMI ≤25 or absence of peritoneal carcinosis showed higher median PFS values compared to those observed in women with opposite features: 45 versus 17 months for BMI (95% CI, 34–55 *vs*. 12–28; log-rank p-value <0.0001) or absence of peritoneal carcinosis (95% CI, 34–53 *vs*. 12–24; log-rank p-value <0.0001).

A further multivariate analysis conducted on validation cohort confirmed that low BTN3A1 concentrations (≤4.75 ng/ml) in plasma, age at diagnosis ≤60 years, and absence of peritoneal carcinosis are independent prognostic factors for a longer PFS in women with advanced HGSOC (**Table 3**). Lastly, also in this cohort, two-factor multivariate analyses suggested that baseline serum CA125 levels >401 U/ml and each soluble protein above respective concentration cutoff were covariates associated with shorter PFS and unfavorable clinical outcome (data not shown).

**Table 3:** Univariate and multivariate analysis of biomarkers and other factors for PFS in the validation cohort.

Factor/biomarker	Univariate Cox regression		Multivariable Cox regression	
	HR (95% CI)	p-value	HR (95% CI)	p*-*value
Age at diagnosis (>60 *vs*. ≤60 years)	5.64 (2.03–15.6)	0.0002	8.12 (2.24–29.5)	0.001
Serum CA125 (>401 *vs*. ≤401 U/ml)	4.91 (1.66–14.5)	0.002	–	NS
BMI (>25 *vs*. ≤25 kg/m^2^)	3.64 (1.47–9.03)	<0.0001	–	NS
Peritoneal carcinosis (yes *vs*. no)	5.86 (1.93–17.8)	<0.0001	12.7 (3.65–44.2)	0.0001
sPD-L1 (>0.42 *vs*. ≤0.42 ng/ml)	2.11 (1.01–4.42)	<0.0001	–	NS
sPD-1 (>2.48 *vs*. ≤2.48 ng/ml)	2.62 (1.13–6.07)	0.003	–	NS
sBTN3A1 (>4.75 *vs*. ≤4.75 ng/ml)	4.30 (1.55–11.9)	<0.0001	4.47 (1.30–15.3)	0.02
pan-sBTN3As (>13.06 *vs*. ≤13.06 ng/ml)	2.38 (0.63–9.07)	0.004	–	NS
sBTN2A1 (>5.59 *vs*. ≤5.59 ng/ml)	2.25 (1.01–4.98)	0.0004	–	NS
sBTLA (>2.78 *vs*. ≤2.78 ng/ml)	2.16 (0.71–6.55)	0.0004	–	NS

BMI, body mass index; CA125, cancer antigen 125; HR, hazard ratio; NS, not significant.

## Discussion

Scientific research is continuously looking for new prognostic indicators able to predict patient survival, enhancing the therapy efficacy. Due to difficulty detected in early detection of OC, the identification of specific biomarkers could improve disease management and provide information helpful for predicting prognosis (30).

Among the numerous investigated biomarkers, CA125, also known as carbohydrate antigen 125, often considered the “gold standard,” has proven to be the most significant indicator involved in screening, detection, management, and survival of OC (31). Serum CA125 levels are measured before surgery in women diagnosed or with suspected diagnosis of OC. Approximately 80% of women affected by epithelial OC show high serum CA125 levels at diagnosis (normal range <35 U/ml) (32). High serum CA125 levels, related to tumor burden and FIGO stages (33), were detected in 50% of early stage disease and 92% of advanced tumors (34). However, several physiological and non-physiological factors affect normal serum CA125 levels, including premenopause, pregnancy (35), menstruation, smoking (34), old age, endometriosis (36), and several malignant conditions, such as breast cancer (37), mesothelioma (38), gastric cancer (39), non-Hodgkin’s lymphoma (40), heart failure (41), and liver cirrhosis (42). In addition, BMI is also positively correlated with CA125 levels, and excess adipose tissue has been shown to lead to increased CA125 levels (34).

In the last years, the ability of PD-L1 and PD-1 to act as a marker for clinical outcome was evaluated by several studies (43), which demonstrated the association between their high expression and poor prognosis in patients harboring different tumors (44–46), including OC (47, 48). However, the prognostic value of tumor PD-L1/PD-1 is still controversial and has not been fully clarified yet in OC (49, 50). In addition, the evaluation of PD-L1/PD-1 expression in primary tumor does not always provide information about the evolution of metastatic disease, since these proteins are dynamic biomarkers (22).

Recently, several studies highlighted an association between poor prognosis and high plasma PD-1 and PD-L1 concentrations in different tumors (21, 25, 26, 28), although this correlation has been little studied, to date, in OC patients (51, 52).

Furthermore, in recent years, our research group analyzed the plasma levels of other immunomodulatory proteins, such as BTLA and butyrophilins, in individuals with different cancers (11, 21, 22).

Since several studies demonstrated the prognostic impact of pretreatment serum CA125 levels in predicting the optimal treatment strategy, clinical outcome, and survival in OC (27), our investigation focused on the search for potential correlations between serum CA125 and circulating levels of immunomodulatory molecules, such as sPD-L1, sPD-1, sBTN3A1, pan-sBTN3As, sBTN2A1 and sBTLA, in 100 advanced HGSOC women. In particular, the aim of our study was to investigate if soluble forms of these immune checkpoints may enhance prognostic power of CA125 in advanced HGSOC.

A survival analysis by Kaplan–Meier curves highlighted that plasma concentrations of each immunoregulatory protein were inversely correlated with PFS of advanced HGSOC patients, allowing to divide them into two subgroups on the basis of a longer (≥30 months) versus shorter PFS (<30 months). A benefit in median PFS ranging from 6 to 16 months was observed when circulating levels of soluble proteins were below the specific concentration thresholds. This suggests that, in the future, sPD-1, sPD-L1, sBTN3A1, pan-sBTN3As, sBTN2A1, and sBTLA could act as useful biomarkers for predicting survival of women with advanced HGSOC, enabling to improve patient clinical management and adopt personalized therapeutic strategies for some patients.

Additionally, our investigation also assessed the impact of age at diagnosis, serum CA125, baseline BMI, and peritoneal carcinomatosis at onset on survival of advanced HGSOC patients, suggesting the negative effect of age at diagnosis over 60 years, high serum CA125 levels (>401 U/ml), excess body weight (BMI > 25), or presence of peritoneal carcinomatosis on PFS.

Furthermore, a multivariate analysis performed to study the impact of different baseline covariates (circulating immunomodulatory proteins, age at diagnosis, serum CA125, BMI, and peritoneal carcinomatosis) on PFS revealed that only the plasma concentration of sBTN3A1>4.75 ng/ml, age at diagnosis >60 years, and presence of peritoneal carcinomatosis were independent prognostic factors for a shorter PFS (<30 months) of advanced HGSOC women. This suggests that circulating sBTN3A1 levels, age at diagnosis, and presence/absence of peritoneal carcinomatosis rather than serum CA125 levels should be considered before starting the therapeutic treatment in advanced HGSOC patients.

BTN3A1 showed a significant immunoregulatory function exerted through modulation of the anti-tumor immune response and activation of γδ T cells (53, 54). Since BTN3A1 is highly expressed in malignant tissues of HGSOC compared to benign ovarian tumors and normal tissues and is associated with poor clinical outcome (53, 55), our results about the correlation between high plasma levels of its soluble form and unfavorable prognosis in advanced HGSOC women are consistent with what was expected. In addition, targeting of BTN3A1 has been shown to transform BTN3A1 from an immunosuppressive to an immunostimulatory molecule, by inducing γδ T-cell-mediated anti-tumor cytotoxicity, resulting in the killing of specific tumor cells by γδ T cells. This may represent an interesting strategy for the treatment of tumors resistant to immunotherapy (55).

Finally, additional two-factor multivariate analyses highlighted that circulating levels of each immunomodulatory protein (sPD-1, sPD-L1, sBTN3A1, pan-sBTN3As, sBTN2A1, or sBTLA) were individually associated with serum CA125 levels, suggesting that contemporary measurement of both biomarkers than CA125 only could strengthen the prognostic power of serum CA125 in predicting PFS of advanced HGSOC women.

Although this investigation provides significant and useful information to current knowledge in the field, it presents some potential limitations, including the relatively limited number of analyzed patients in the leading cohort, the lack of a sufficiently large validation set able to test each new putative biomarker (despite data shows a good statistical power), and the potential interference of the plasma matrix during the dosage in plasma of immune checkpoints through ELISA assays (although a one-fifth dilution of the samples seems to overcome this problem). In addition, a larger number of studies are needed to deeply investigate the releasing mechanisms (to date, unknown) of the soluble form of each immunoregulatory protein from tumors and/or stromal cells.

## Data availability statement

The raw data supporting the conclusions of this article will be made available by the authors, without undue reservation.

## Ethics statement

This study was reviewed and approved by Comitato Etico Palermo 1, University-affiliated Hospital A.O.U.P. “P. Giaccone” of Palermo (Italy)- (Protocol “TIC-OC v.1.1”). The patients/participants provided their written informed consent to participate in this study.

## Author contributions

Conceptualization: DF, JLI, AR, VB and VC. Sample collection and investigation: DF, CB, LRC, SC, MCDD, CF, MCL, LM, UR, SV, GP and VC. Experimental analysis: DF and JLI. Data curation and analysis: DF, CB and LRC, MCDD, SC, CF, MCL, LM and TDBR. Writing: DF, CB and LRC. Critical revision of the manuscript: DF, JLI, AR, VB, VC, SV and GP. Supervision: JLI, AR, VB and DF. The figures of the manuscript were conceived and designed by DF, CB, LRC, UR and TR. The tables were conceived and designed by DF, CB, LRC, UR, LM and TR. Literature data were acquired and analyzed by DF, CB, LRC, LM, UR and TR. All authors contributed to the article and approved the submitted version.

## Acknowledgments

All authors thank Dr. Chiara Drago for the language English revision. All authors thank the following nurses for the blood draws: Accurso Gaspare, Argentano Salvatore, Basile Anna, Curatolo Giovanna, D’Angelo Davide, Fricano Emanuela, Grizzanti Elisa, La Corte Carolina, Laganà Lucio, Marino Giuseppina, Martorana Eleonora, Pellerito Ilenia, and Sciortino Melania.

## Conflict of interest

JLI is cofounder of PanCa Therapeutics and PredicitngMed.

The remaining authors declare that the research was conducted in the absence of any commercial or financial relationships that could be construed as a potential conflict of interest.

## Publisher’s note

All claims expressed in this article are solely those of the authors and do not necessarily represent those of their affiliated organizations, or those of the publisher, the editors and the reviewers. Any product that may be evaluated in this article, or claim that may be made by its manufacturer, is not guaranteed or endorsed by the publisher.
