# Supplementary material for: Can circulating PD-1, PD-L1, BTN3A1, pan-BTN3As, BTN2A1 and BTLA levels enhance prognostic power of CA125 in patients with advanced high-grade serous ovarian cancer?
Source: Front Oncol. 2022 Sep 21;12:946319. doi: 10.3389/fonc.2022.946319 (PMC9532861; doi:10.3389/fonc.2022.946319)
Supplement: Supplementary file 1 [file DataSheet_1.docx]

**Supplementary Tables**

**Table S1. Characteristics of ELISAs for sPD-L1, sPD-1, pan-sBTN3As, sBTN3A1, sBTN2A1, and sBTLA.**

|  | PD-L1 | PD-1 | pan-BTN3As* | BTN3A1* | BTN2A1 | BTLA |
| --- | --- | --- | --- | --- | --- | --- |
| Coating Ab | α-PD-L1 1.8 +  α PD-L1 2.1 | α-PD-1 6.4 | α-BTN3A S148 | α-BTN3A1 S240 | α-BTN2A1 8.16 | α-BTLA 75.2 |
| Detection Ab (biotinylated) | Α-PD-L1 1.3.1 | α-PD-1 3.1 | α-BTN3A  103.2 | α-BTN3A  103.2 | α-BTN2A1 4.15  + α-BTN2A1 5.28 | α-BTLA 7.1  +  α-BTLA 8.2 |
| Detection limit (pg/ml) | 20 | 50 | 100 | 100 | 30 | 200 |

* Three isoforms of BTN3A are identified (A1, A2, A3). Among available monoclonal antibodies to BTN3A, one is specific for A1 (α-BTN3A1 S240). Coating with α-BTN3A1 S240 allows specific assay of the A1 isoform, whereas the couple of antibodies α-BTN3A S148 and α-BTN3A 103.2 allows simultaneous detection of all 3 forms (Pan-BTN3A assay). It is however noteworthy that BTN3A concentrations obtained with the Pan-BTN3A assay are only indicative since the range used in the assay is pure BTN3A1. BTN3A concentrations should therefore be expressed as pg/ml « equivalent BTN3A1».

**Table S2. Two-factor multivariate analyses of each circulating immune checkpoint with serum CA125 for PFS in advanced HGSOC patients.**

| **Biomarker** | **Multivariable Cox Regression** | |
| --- | --- | --- |
|  | **HR (95% CI)** | ***p-*Value** |
| sPD-1  (>2.48 *vs* ≤2.48 ng/mL)  Serum CA125  (>401 *vs* ≤401 U/ml) | 1.56 (1.00-2.44)  1.70 (1.13-2.58) | 0.05  0.01 |
| sPD-L1  (>0.42 *vs* ≤0.42 ng/mL)  Serum CA125  (>401 *vs* ≤401 U/ml) | 2.95 (1.81-4.82)  1.67 (1.10-2.54) | <0.0001  0.01 |
| sBTN3A1  (>4.75 *vs* ≤4.75 ng/mL)  Serum CA125  (>401 *vs* ≤401 U/ml) | 2.77 (1.76-4.38)  1.76 (1.16-2.67) | <0.0001  0.008 |
| pan-sBTN3As  (>13.06 *vs* ≤13.06 ng/mL)  Serum CA125  (>401 *vs* ≤401 U/ml) | 2.54 (1.62-3.97)  1.74 (1.15-2.64) | <0.0001    0.009 |
| sBTN2A1  (>5.59 *vs* ≤5.59 ng/mL)  Serum CA125  (>401 *vs* ≤401 U/ml) | 1.86 (1.18-2.94)  1.70 (1.13-2.57) | 0.007  0.01 |
| sBTLA  (>2.78 *vs* ≤2.78 ng/mL)  Serum CA125  (>401 *vs* ≤401 U/ml) | 2.16 (1.39-3.35)  1.72 (1.13-2.61) | 0.0006  0.01 |

Abbreviations: BMI, Body Mass Index; CA125, Cancer antigen 125; HR, Hazard Ratio; NS, Not Significant.

**Supplementary file 1. Linearity and specificity of used ELISA tests**

| **[sPD-1] ng/mL** | **O.D.** |
| --- | --- |
| **5.00** | 2.073 |
| **1.00** | 0.609 |
| **0.50** | 0.322 |
| **0.10** | 0.058 |
| **0.05** | 0.025 |
| **0.01** | 0.003 |
| **0.00** | 0.000 |
| **R²** | 0.9980 |

| **[sPD-L1] ng/mL** | **O.D.** |
| --- | --- |
| **2.00** | 3.006 |
| **1.00** | 1.660 |
| **0.50** | 0.870 |
| **0.25** | 0.438 |
| **0.10** | 0.172 |
| **0.02** | 0.036 |
| **0.00** | 0.000 |
| **R²** | 0.9970 |

| **[Pan-sBTN3As] ng/mL** | **O.D.** |
| --- | --- |
| **8.00** | 0.759 |
| **4.00** | 0.401 |
| **1.00** | 0.106 |
| **0.50** | 0.058 |
| **0.10** | 0.021 |
| **0.05** | 0.000 |
| **0.00** | 0.000 |
| **R²** | 0.9999 |

| **[sBTN3A1] ng/mL** | **O.D.** |
| --- | --- |
| **8.00** | 1.040 |
| **4.00** | 0.576 |
| **1.00** | 0.159 |
| **0.50** | 0.081 |
| **0.10** | 0.017 |
| **0.05** | 0.009 |
| **0.00** | 0.000 |
| **R²** | 0.9955 |

| **[sBTN2A1] ng/mL** | **O.D.** |
| --- | --- |
| **2.00** | 2.173 |
| **1.00** | 1.188 |
| **0.50** | 0.611 |
| **0.25** | 0.308 |
| **0.125** | 0.160 |
| **0.06** | 0.086 |
| **0.03** | 0.052 |
| **0.00** | 0.000 |
| **R²** | 0.9998 |

| **[sBTLA] ng/mL** | **O.D.** |
| --- | --- |
| **8.00** | 1.801 |
| **4.00** | 0.980 |
| **2.00** | 0.510 |
| **1.00** | 0.259 |
| **0.5** | 0.131 |
| **0.25** | 0.067 |
| **0.00** | 0.000 |
| **R²** | 0.9990 |

**Supplementary file 2. Experimental protocol for the used ELISA assays**

- All steps of four ELISA tests are run at Room Temperature (RT).
- Before starting assay, all plasma samples were diluted 1/5 on a final volume of 100 µL (20 µL plasma + 80 µL of dilution buffer) for each well, in order to make negligible the interference of the plasma matrix.
- The dilution buffer used to dilute the plasma samples consists of Tris/NaCl/BSA/trehalose/thimerosal.
- The plates are coated overnight with the antibody selected for capture diluted in Tris buffer and then blocked with Tris/BSA buffer.
- For the dosage, the specific recombinant protein (PD-L1/PD1/BTN3A1/pan-BTN3As) is placed in the plate with an associated control (dilutions are made in the dilution buffer described above). A negative control (named “white”) is used. Samples to be tested are incubated for 3 hours at RT.
- Wash 5 times in PBS-Tween buffer.
- A biotinylated antibody (diluted in Tris buffer /rabbit serum) is added to form the “sandwich”. Incubate for 30 min at RT.
- Wash 5 times in PBS-Tween buffer.
- Avidin-peroxidase (HRP) conjugate is added. Incubate for 15 min at RT.
- Add the TMB (3,3′,5,5′-Tetramethylbenzidine) substrate. Incubate for 15 min at RT in the dark.
- The reaction is stopped with H_2_SO4 and OD (optical density) read at 450 nm.
